# The phenotypic plasticity of an evolving digital organism

**DOI:** 10.1098/rsos.220852

**Published:** 2022-09-14

**Authors:** Miguel A. Fortuna

**Affiliations:** Computational Biology Lab, Estación Biológica de Doñana (EBD), Spanish National Research Council (CSIC), Seville, Spain

**Keywords:** artificial life, digital evolution, evolutionary innovation, fitness cost, polyphenism

## Abstract

Climate change will fundamentally reshape life on Earth in the coming decades. Therefore, understanding the extent to which species will cope with rising temperatures is of paramount importance. Phenotypic plasticity is the ability of an organism to change the morphological and functional traits encoded by its genome in response to the environment. I show here that plasticity pervades not only natural but also artificial systems that mimic the developmental process of biological organisms, such as self-replicating and evolving computer programs—digital organisms. Specifically, the environment can modify the sequence of instructions executed from a digital organism’s genome (i.e. its transcriptome), which results in changes in its phenotype (i.e. the ability of the digital organism to perform Boolean logic operations). This genetic-based pathway for plasticity comes at a fitness cost to an organism’s viability and generation time: the longer the transcriptome (higher fitness cost), the more chances for the environment to modify the genetic execution flow control, and the higher the likelihood for the genome to encode novel phenotypes. By studying to what extent a digital organism’s phenotype is influenced by both its genome and the environment, I make a parallelism between natural and artificial evolving systems on how natural selection might slide trait regulation anywhere along a continuum from total environmental control to total genomic control, which harbours lessons not only for designing evolvable artificial systems, but also for synthetic biology.

## 1. Introduction

Phenotypic plasticity is the ability for organisms with the same genotype to produce distinct, but repeatable, phenotypes under different environmental conditions [1].

Some remarkable examples are predator avoidance [2,3], insect polyphenism [4,5], osmoregulation in fish [6,7] and the timing of metamorphosis in amphibians [8,9]. In contrast to the concept of canalization, introduced by Waddington [10] to explain the tendency of organisms to produce always the same phenotype despite changes in genotype and environment, plasticity is not a property of the developmental process but of the genotype. The genotype becomes hence a repertoire of potential developmental outcomes [11]. But in spite of its importance as an adaptive force shaping biodiversity, phenotypic plasticity has been given little consideration in evolutionary theory. Yet, some authors have recently stressed that phenotypic plasticity should be seen as one important element in an extended synthesis of evolution [12].

Phenotypic plasticity may or may not have a genetic basis. Plasticity without a genetic basis can be considered a by-product of the physics and chemistry of the developmental process. For example, temperature affects the underlying chemical and metabolic processes of development directly, without the intervention of any genetic mechanism [13]. Heritable epigenetic variation (e.g. DNA methylation) can also lead to inducible and persistent developmental changes [14]. By contrast, long before discovering the mechanisms inducing this non-genetic variation, Bradshaw [15] suggested that plasticity is a trait that can have a genetic basis and that therefore can evolve. Since then, much work focuses on changes in gene expression patterns under different environmental conditions (i.e. genes for plasticity, see e.g. [7]), highlighting the potential for what has been termed reaction norms [16] and the plasticity of gene expression [17].

Woltereck [18] coined the term 'reaction norm' to describe the relationship between environmental variation and phenotypic variation. For continuous traits, the reaction norm is a function usually represented as a line on a graph that plots a phenotype against an environmental factor [19]. For discrete traits, phenotypic variation results in alternative phenotypes known as polyphenisms [4]. The shape of the reaction norm (e.g. plastic responses to climate change) is a consequence of changes in transcription induced by the environment [20]. Some of these changes exhibit genotype-by-environment interaction, involving an environmental plastic genetic architecture of gene expression [21,22].

Changes in transcription (e.g. an increase in the level of gene expression) can impact the fitness of an organism. There is evidence that transcription is costly, and that this cost is important during evolution [23]. But, understanding the costs of transcription requires us to distinguish between the cost of the phenotype and the cost of plasticity [24,25]. The cost of the phenotype refers to the fitness reduction resulting from having one phenotype rather than another in a particular environment [24]. By contrast, the cost of plasticity refers to the fitness reduction a highly plastic genotype pays relative to a less plastic genotype [26]. Thus, whereas phenotype costs are environment dependent, costs of plasticity are global, that is, they exist in all environments.

Quantifying the cost of plasticity requires a biologist to meet the challenge of finding distinct genotypes that have the same phenotype in a particular environment but differ in their plastic responses to a variable environmental factor. In well-studied microbes, most traits are plastic and many are amenable to environmental manipulations using laboratory culture techniques (e.g. [27]). One advantage of microbial experimental evolution is that it can be performed for many generations, with large populations, and with replication (e.g. [28]). Nevertheless, the genetic mechanisms regulating their phenotypes and their plasticity—such as structural and regulatory genes—are known only in a few cases.

**Figure 1.**
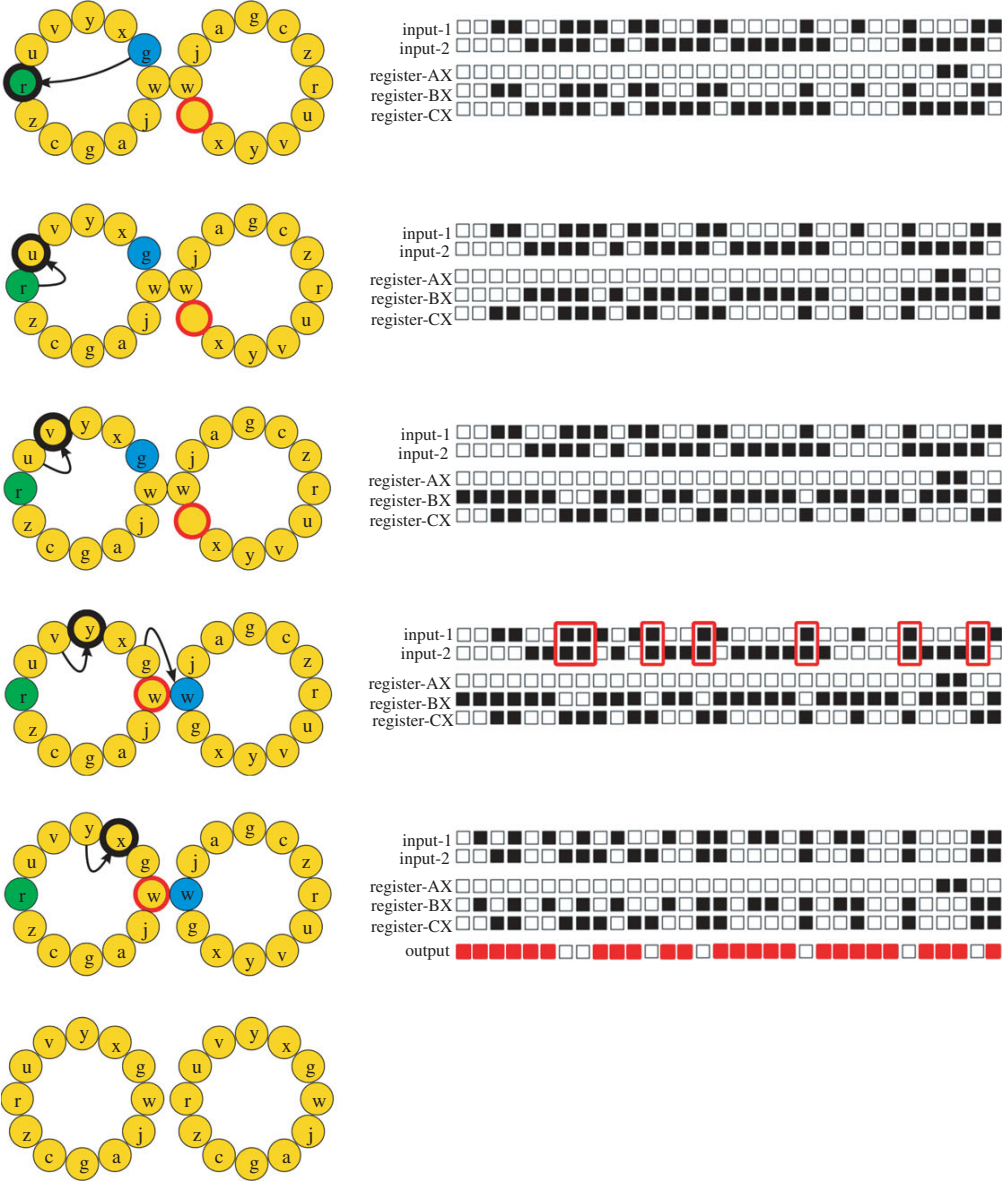
Cartoon depicting the last stages of the replication cycle of a digital organism. The genotype of the parent organism is represented on the left as a circular set of 12 instructions (depicted as letters). The growing offspring is represented to the right of its parent. The bits of the binary numbers stored in the buffers and registers (shown before the execution of the corresponding instruction) are represented as a sequence of 32 boxes, one for each bit (a black box if it is 1 and a white box if it is 0). (1) *swap (r)* swaps the contents of the BX register with its complement (CX register); (2) *nand (u)* reads the contents of the BX and CX registers and performs a bitwise *nand* operation on them, placing the result of this operation in the BX register; (3) *h-copy (v)* reads in the contents of the organism’s memory at the position of the red-head (blue), copies that to the position of the write-head (red), and advances both heads (the read-head to the first position of the offspring, and the write-head to the first position of the parent); (4) *input–output (y)* takes the contents of the BX register and outputs it, checks it for any logic operation that may have been performed on the two input numbers (highlighted in red), and places a new input number into the BX register and the first input buffer (after moving its value to the second input buffer); (5) *h-divide (x)* divides off a finished offspring located between the read-head and the write-head. The genome of this digital organism encoded the NAND logic operation (i.e. for each bit pair, the result is 0 (white box) if and only if the two bits are 1, and 1 otherwise (red box)).

The concept of digital twins as software replicas of biological processes [29] may help us understand the mechanisms responsible for phenotypic plasticity, their associated costs and evolutionary implications. It may also be a powerful tool to compare and contrast natural and artificial evolving systems, including the extent to which natural systems are more sensitive to genetic changes induced by the environment. In this paper, I use self-replicating computer programs that mutate and evolve within a user-defined computational environment as a digital twin. Specifically, we focus on the digital organisms implemented in the Avida software platform [30] because their phenotypes result from the execution flow of the computer instructions harboured in their genomes—a mechanism analogous to the developmental process of biological organisms (figure 1). Interestingly, the genetic language of a digital organism contains instructions that can modify the execution flow of an organism’s genome in response to the environment, a mechanism analogous to the transcriptional regulation of biological organisms. In addition, the environmental sensitivity of these regulators might overcome the constraints imposed by the genetic system to produce canalization. Therefore, phenotypic plasticity might be more prevalent in artificial evolving systems than expected.

In this study, I quantify to what extent a digital organism’s phenotype is influenced by both its genome and the environment, meaning that selection might slide trait regulation anywhere along a continuum from total environmental control to total genomic control. To this end, I first quantify the prevalence of plasticity in digital organisms. Second, I identify the genetic basis of phenotypic plasticity and disentangle the genetic and non-genetic mechanisms responsible for plasticity. Third, I quantify the fitness costs of plasticity in terms of reduced organism’s viability and longer generation times and explore the reasons underlying the trade-off between fitness costs and phenotypic plasticity. Lastly, I compute the uncertainty of finding a particular phenotype in a novel environment as a function of the mechanism responsible for plasticity. Although my focus here is on how phenotypic variation arises (as opposed to a focus based on the consequences of that variation), I also discuss the fitness implications of plasticity under different selective pressures.

## 2. Methods

### 2.1. Avida as digital evolution software platform

Digital evolution is a form of evolutionary computation in which self-replicating computer programs—digital organisms—evolve within a user-defined computational environment. Avida is the most widely used software platform for research in digital evolution [30]. It satisfies three essential requirements for evolution to occur: replication, heritable variation and differential fitness. The latter arises through competition for the limited resources of memory space and central processing unit, CPU time. A digital organism in Avida consists of a sequence of instructions—its genome or genotype—and a virtual CPU, which executes these instructions. Variation that arises through mutation generates competition among genotypes as the population grows and uses up available resources. The genotype that is fittest, in the sense of being the faster replicator under the prevailing conditions of growth (or that encodes a phenotype required to metabolize a nutrient in specific environments, see below), comes to dominate the population through natural selection. This leads to a replacement of one genotype by another and so to a change in the genetic makeup of the population. For a more detailed description of Avida and the parallelism between natural and artificial evolving systems, see [31].

### 2.2. The genotype of a digital organism

The genome of a digital organism is a circular sequence of instructions taken from a 26-instruction alphabet [30,31]. Some of these instructions are involved in copying an organism’s genome, which is the only way the organism can pass on its genetic material to future generations. To reproduce, a digital organism must copy its genome instruction by instruction into a new region of memory through a process that may lead to errors (i.e. mutations). A mutation occurs when an instruction is copied incorrectly, and is instead replaced in the offspring genome by an instruction chosen at random (with a uniform distribution) from a set of possible instructions. Some instructions are required for a successful replication (i.e. organism’s viability), whereas others are required to manipulate binary numbers taken from the environment through input–output instructions (such as addition, multiplications and bit-shifts). The genome of a digital organism can harbour one or several input–output instructions that can be executed either only once or many times during the time it takes to generate an offspring. This means that the organism can take input numbers from the environment more than once before replicating and can compute the result of more than one logic operation (see below).

### 2.3. The phenotype of a digital organism

The genetic language of Avida contains instructions for copying an organism’s genome as well as for storing and manipulating 32-bit binary numbers in buffers (input-1 and input-2), stacks, and registers (AX, BX and CX; see [30,31] for more details). One of these instructions—the input–output instruction—plays a key role in determining an organism’s phenotype, although other instructions are important too (figure 1). When an input–output instruction is executed by an organism’s CPU, the organism outputs the 32-bit binary number stored in its BX register (i.e. the default memory space that a digital organism uses for storing a single 32-bit binary number), checking for any logic operation that may have been performed on the two binary numbers previously stored in its input buffers (i.e. storage spaces that a digital organism uses to receive numbers). For example, for the output to be the result of applying the logic operation NAND on the two 32-bit binary numbers stored in its input buffers, each bit of the output number should be 0 when and only when the two bits of the input bit pair are 1, and 1 otherwise. Next, the input–output instruction places a new random 32-bit binary number into its BX register (a number that is also stored in its input-1 buffer after replacing the number stored in its input-2 buffer by the number previously stored in its input-1 buffer).

The phenotype of an organism is defined by the combination of the following nine logic operations that it can compute when executing input–output instructions: *NOT*, which returns 1 at a bit position if the input is 0 at that bit position, and 0 if the input is 1; *NAND*, which returns 0 if and only if both inputs at the corresponding bit positions are 1 (otherwise it returns 1); *AND*, which returns 1 if and only if both inputs are 1 (otherwise it returns 0); *OR_N* (or-not), which returns 1 if for each input bit pair one input bit is 1 or the other is 0 (otherwise it returns 0); *OR*, which returns 1 if either the first input, the second input, or both are 1 (otherwise it returns 0); *AND_N* (and-not), which only returns 1 if for each bit pair one input is 1 and the other input is 0 (otherwise it returns 0); *NOR* (not-or), which returns 1 only if both inputs are 0 (otherwise it returns 0); *XOR* (exclusive or), which returns 1 if one but not both of the inputs are 1 (otherwise it returns 0); *EQU* (equals), which returns 1 if both bits are identical, and 0 if they are different.

### 2.4. The phenotypic plasticity of a digital organism

I hereby refer to phenotypic plasticity as the ability of a digital organism’s genome to perform distinct logic operations in different environments. The environment may modify the phenotype encoded by an organism’s genome every time the organism’s CPU executes an input–output instruction. Since this instruction takes the content of the organism’s BX register and outputs it, checking it for any logic operations that may have been performed on the two 32-bit binary numbers stored in its input buffers, an organism’s phenotype depends on the content of the organism’s BX register when an input–output instruction is executed, and this content depends initially—and later on, every time an input–output instruction is executed—on the environment.

The genome of a digital organism can harbour one or several input–output instructions that can be executed either only once or many times during the time it takes to generate an offspring. This means that an organism can take input numbers from the environment more than once before replicating and can compute the result of more than one logic operation. Note that a particular environment is associated with a unique set of 32-bit binary numbers that can be supplied to the BX register (i.e. an organism’s genome will always encode the same phenotype in the same environment because the same random sequence of binary input numbers will be provided). Therefore, two identical genomes may encode different phenotypes in different environments because, although they could execute the same sequence of instructions, their BX registers might contain different binary numbers and hence, the input–output instruction would output different binary numbers that might not be the result of computing the same logic operation.

An organism can further change the content of its BX register by executing other instructions (noted below in italics) that might be contained in its genome. This additional change can happen by (i) performing a bitwise NAND operation on the BX and CX registers and placing the result in the BX register (*nand*), (ii) adding (substracting) to its content the content of the CX register (*add* and *sub*, respectively), (iii) incrementing (decrementing) by one its content (inc and *dec* instructions, respectively), (iv) swapping its content with the content of the CX register (*swap*), (v) moving numbers between the active stack (i.e. a storage space that a digital organism uses for storing numbers) and the BX register (*pop* and *push*, in addition to *swap-stk* that toggles the active stack in use) and (vi) shifting all of the bits in the BX register by one to the left (*shift-l*) or right (*shift-r*). Therefore, two different genomes in the same environment (i.e. storing initially the same content in their BX registers) might encode distinct phenotypes if one of them executes any of these instructions before an input–output instruction.

Changing the content of an organism’s BX register, either by placing a new input binary number through the input–output instruction or through the manipulation of its content carried out by any of the above instructions, constitutes the non-genetic mechanism underlying phenotypic plasticity in digital organisms.

**Figure 2.**
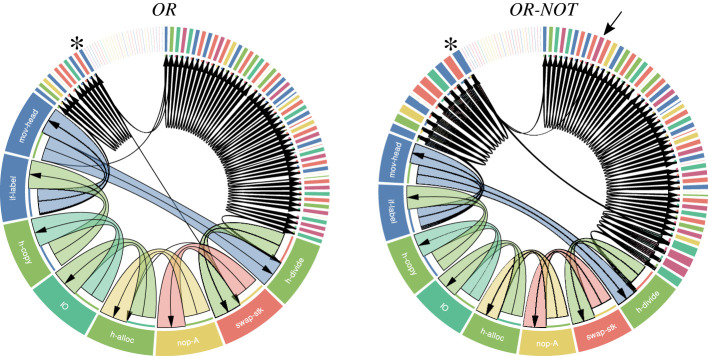
Phenotypic plasticity resulting from changes in the transcription of a digital organism’s genome. The same genome encodes two distinct phenotypes in different environments by modifying the sequence of instructions that were actually executed. The resulting transcriptomes are represented as chord diagrams: the one on the left computed the logic operation *OR*, and the one on the right the logic operation *OR-NOT*. The instructions of the 100-length genome that were actually executed are represented as segments around the circle. The size of each segment is proportional to the number of times that the instruction was executed, and a label indicating the name of the instruction was included within that genome’s slice for the most frequently executed ones (gaps between segments indicate parts of the genome that were not executed). Colours of the segments indicate the type of the instruction (yellow for no-operations, blue for flow control operations, red for math operations, green for operations involved in replication, and turquoise for the input–output (IO) operation). An arrow from one segment to another represents the order at which instructions were executed, and its colour corresponds to that of the latest instruction executed. The first instruction of the genome that is executed is placed at the top centre of the chord diagram. The length of the transcriptome computing *OR-NOT* is more than twice the length of the transcriptome computing *OR* (instructions at the top-left and right part of the right chord were executed more times). Besides executing more instructions, different paths in the execution flow were traced in each environment. The instruction *jump-head* (marked with an asterisk) was executed three times during the replication cycle in both environments. This instruction reads the value of the CX register and moves the instruction pointer by that fixed amount through the organism’s memory. The next instruction to be executed depends on the new location of the instruction pointer. For example, if it is placed on the *swap-stk* instruction (left), it will switch the active stack in use and hence, modify the result of moving numbers between the BX register and the active stack. On the contrary, if it is placed on the *sub* instruction (right, marked with an arrow), it will read the contents of the BX and CX registers, subtract CX from BX, and place the result of this operation in the BX register. Therefore, in this example phenotypic plasticity results from a genetic-based mechanism.

The phenotype of a digital organism may also be modified through a genetic-based mechanism (figure 2). That is, the content of an organism’s BX register may change the sequence of instructions to be executed by its CPU by (i) skipping the next instruction if the content of the BX register is greater than (if-less) or equal to (if-n-equ) the content of the CX register, (ii) moving the instruction pointer through the organism’s memory by a fixed amount determined by the content of the CX register (*jump-head*) and (iii) moving the flow-head to the memory position specified by the CX register (*set-flow*). I will hereby refer to these instructions as *regulatory* because of their role in shaping the execution flow of an organism’s genome. Therefore, two identical genomes may also encode different phenotypes in different environments because they might execute different sequences of instructions and hence, the input–output instruction would output different binary numbers that might not be the result of computing the same logic operation (figure 2).

### 2.5. The transcriptome of a digital organism

Every time a regulatory instruction is executed (i.e. *if-less*, *if-n-equ*, *jump-head* or *set-flow*), the content of an organism’s BX register may change the sequence of instructions to be executed by the organism’s CPU during the time it takes to generate an offspring (figure 2). This change may imply the execution of instructions that are part of the organism’s genome but never executed, or that of those that may further modify the content of its BX register. It may also imply a change in the number of instructions executed before producing a viable offspring and hence, a change in the organism’s fitness. It might even turn an otherwise viable organism into a non-viable one (i.e. an organism incapable of producing any offspring able to self-replicate). I hereby refer to the sequence of instructions that are actually executed by an organism’s CPU during the time it takes to generate an offspring as the *transcriptome* of a digital organism.

### 2.6. Dataset

I built a database of digital organisms by randomly sampling the genotype space of 100-instruction long genomes encoding the same phenotype in the same single environment. Since I covered the entire phenotype space comprising nine Boolean logical operations (i.e. 2^9^ = 512 distinct phenotypes) and sampled 1000 distinct genomes encoding the same phenotype, the database contained 512 000 distinct genomes [31]. To obtain the phenotypic plasticity of digital organisms, I computed, for each organism in the database, the phenotype encoded by its genome in 1000 distinct environments. To disentangle the genetic and non-genetic mechanisms responsible for plasticity, I traced, for each organism and environment, the sequence of instructions of the genome that were actually executed by an organism’s CPU during the replication process (figure 2).

### 2.7. Statistical analysis

I classified organisms according to the number of distinct phenotypes they encoded (i.e. plastic or non-plastic organisms, if they encoded more than one or the same phenotype, respectively). Changes in transcription mediated by the environment that did not produce changes in the phenotype were also quantified as a source of canalization. That is, I also classified organisms according to the number of distinct transcriptomes they executed (i.e. robust- or sensitive-transcriptome organisms, if they executed the same or more than one transcriptome, respectively) in the environments in which they were viable (i.e. if they were capable of producing an offspring during its replication cycle).

Then, I used a generalized linear model (binomial distribution; link function = logit) to quantify the effect of the sensitivity of an organism’s genome to environmental-mediated changes in transcription (robust- or sensitive-transcriptome organism) on the probability of an organism to show phenotypic plasticity (single- or plastic-phenotype organism). I also used a generalized linear model (binomial distribution; link function = logit) to quantify the effect of genetic-based phenotypic plasticity (i.e. changes in transcription) on the costs to an organism’s viability. I included phenotypic plasticity (single- or plastic-phenotype organisms) of only the sensitive-transcriptome organisms as fixed effect, and the probability for an organism to be viable in all environments as the binary response variable. To quantify the effects of the number of distinct transcriptomes executed by the same genome in different environments and the viability costs of the changes in transcription on the number of distinct phenotypes encoded by that genome in those environments, I used a zero-truncated negative binomial model link function = log. I categorized the costs of changes in transcription on an organism’s viability as a binary variable (zero if the organism was viable in all environments, and one otherwise). I also scaled the logarithm of the number of distinct transcriptomes (mean = 0, standard deviation = 1) to aid the interpretation of both main effects and statistical interaction in my analysis [32]. The effect of the sensitivity of an organism’s genome to environmental-mediated changes in transcription (robust- or sensitive-transcriptome organism) on fitness measured as the number of instructions a digital organism must execute to produce a viable offspring (i.e. the length of the transcriptome) was quantified using a linear model. The effect of the same factor on the number of *input–output* instructions executed during the replication cycle was quantified using a generalized linear model (poisson distribution; link function = log). Lastly, I used a generalized linear model (quasi-binomial distribution; link function = logit) to quantify the effect of the sensitivity of the genome to changes in transcription (zero if an organism executed the same transcriptome in all environments where it was viable and one otherwise) on the uncertainty of computing a logic operation in a particular environment measured as the Shannon entropy (base-2 logarithm). The coefficient estimates of the generalized linear models are on the log odds (binomial) and log scale (zero-truncated negative binomial and poisson), but I explain the results on the original scale (probability and counts, respectively), which is more intuitive to understand. To calculate these probabilities, I applied the inverse logit, 1/(1 + exp ^−*β*^), and the inverse log, exp^*β*^, estimates to the regression models reported in the main text.

## 3. Results

**Figure 3.**
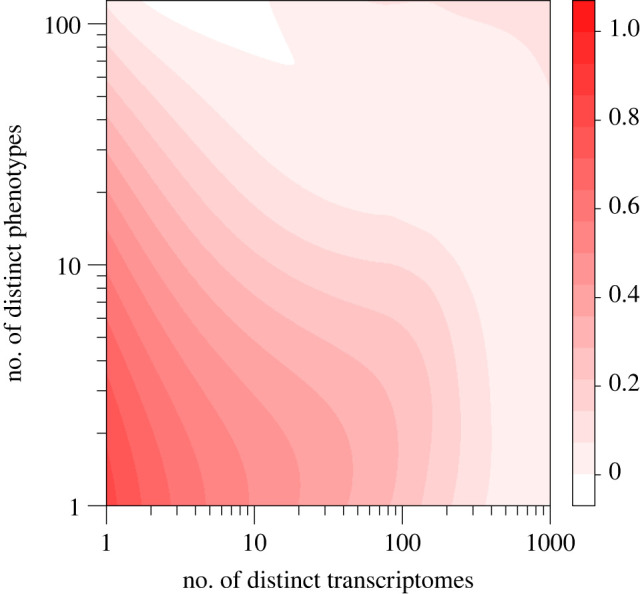
Distribution map of phenotypic plasticity and sensitivity to changes in transcription of a digital organism’s genome. The contour map represents the relationship between the number of distinct phenotypes encoded by an organism’s genome in different environments and the number of distinct transcriptomes executed by the organism holding that genome in those environments. Colour saturation indicates the relative frequency of organisms having a particular combination of number of phenotypes and transcriptomes (the darker the red colour the higher the frequency). Data points (*n* = 8458) are not shown for better visualization. The higher the phenotypic plasticity, the sensitivity to changes in transcription, or both, the fewer the number of organisms having those properties. The genomes of half organisms (52%) encoded a single phenotype and executed the same transcriptome in all environments. Only 3% of genomes encoded the same phenotype in all environments by changing the transcriptome. By contrast, 29% of genomes encoded more than a single phenotype by executing the same transcriptome in all environments. The remaining 16% were plastic-phenotype and sensitive-transcriptome organisms (i.e. phenotypic plasticity with a genetic basis). Organisms encoding many phenotypes by executing many different transcriptomes were very rare (top-right corner).

Only slightly more than half of organisms' genomes (55%) encoded the same phenotype in all environments where they were viable (i.e. non-plastic organisms), and almost all of them (95%) did it by executing the same transcriptome; see figure 3. Phenotypic plasticity with no genetic basis (i.e.distinct phenotypes produced without changing the transcriptome) accounted for most plastic organisms (65%). Yet, the probability for an organism to be plastic was more than twice as high ($1/(1+e^{0.56-2.21})=84\text{\%}$) when the mechanism for plasticity had a genetic basis (i.e. its genome was sensitive to changes in transcription mediated by the environment) than when it had a non-genetic basis ($1/(1+e^{0.56})=36\text{\%}$; table 1). In fact, the number of distinct phenotypes encoded by an organism’s genome across environments was 48% higher when the mechanism for plasticity was genetic than when it was non-genetic (e^1.04+0.66^ = 5.5 and e^1.04^ = 2.8, respectively; table 1).

**Table 1.** Changes in transcription mediated by the environment. Results of the generalized linear models used to quantify the effect of the sensitivity of a digital organism’s genome to environmental-mediated changes in transcription on the probability of an organism’s genome to encode a plastic phenotype (left), the number of distinct phenotypes encoded (middle) and the uncertainty of computing a logic operation in a randomly chosen environment (right).

	probability of plasticity			no. of phenotypes			phenotypic uncertainty		
predictors	log-odd	CI (95%)	*p*-value	log-odd	CI (95%)	*p*-value	log-odd	CI (95%)	*p*-value
(intercept)	−0.56	−0.57 to − 0.55	<**0.001**	1.04	1.04–1.04	<**0.001**	2.30	2.30 to 2.31	<**0.001**
transcriptome (sensitive)	2.21	2.20 to 2.23	<**0.001**	0.66	0.66–0.66	<**0.001**	−1.43	−1.44 to −1.43	<**0.001**
observations	510 830			510 830			808 981		

Some genomes encoded non-viable organisms in at least one environment (15%). This might influence the results of the logistic regression model reported above (i.e. the higher the number of environments where the genomes of viable organisms were tested, the larger might be the number of distinct transcriptomes executed and phenotypes encoded). To rule this possibility out, I repeated the analysis by considering only the genomes encoding viable organisms in all environments (85%). The results did not change qualitatively (see Data availability).

Phenotype does not affect an organism’s viability in this study because there is no selective pressure favouring one phenotype over another (i.e. the faster replicator will dominate the population). If a digital organism is viable is one environment (i.e. it can produce an offspring), there is no reason for the organism to not be viable in any other environment unless its transcriptome changes. That is, if the content of the BX register does not change the instructions that a digital organism executes during its replication cycle (i.e. if there is no genetic basis for plasticity), the same sequence of instructions will always be executed and the digital organism will never enter in an infinite execution loop that might jeopardize its viability. Therefore, the organisms excluded from the previous analysis were all sensitive-transcriptome organisms. This means that the genetic basis of phenotypic plasticity comes at a cost to an organism’s viability. In fact, the probability for a sensitive-transcriptome organism to be viable in all environments is lower ($1/(1+e^{0.92+0.34})=22\text{\%}$) when its genome encoded a plastic phenotype than when it encoded a single-phenotype organism ($1/(1+e^{0.92})=29\text{\%}$; table 2). That is, when a change in transcription does not imply a change in phenotype, the probability for a genome to encode a viable organism is higher than when it does.

**Table 2.** Costs of plasticity. Results of the models used to quantify (from top to bottom): (i) the effect of phenotypic plasticity on the probability for a digital organisms to be viable in all environments (logistic model, link function = logit); (ii) the cost of transcription and number of distinct transcriptomes (scaled log) on the number of distinct phenotypes (zero-truncated negative binomial model, link function = log); (iii) the phenotypic plasticity and sensitivity of the genome to changes in transcription on the length of the transcriptome (linear model); and (iv) the sensitivity of the genome of plastic organisms to changes in transcription on the number of *input–output* instructions executed during the replication cycle (poisson model, link function = log).

	probability for an organism to be viable		
predictors	log-odd	CI (95%)	*p*-value
(intercept)	−0.92	−0.95 to −0.88	<**0.001**
phenotype (plastic)	−0.34	−0.38 to −0.30	<**0.001**
observations	95 676		
	no. of distinct phenotypes		
predictors	log	CI (95%)	*p*-value
(intercept)	2.15	2.13 to 2.17	<**0.001**
transcriptomes	−0.04	−0.06 to −0.03	<**0.001**
cost (yes)	−0.56	−0.58 to −0.54	<**0.001**
transcriptomes : cost	0.33	0.31 to 0.34	<**0.001**
observations	80 333		
	transcriptome length		
predictors	estimate	CI (95%)	*p*-value
(intercept)	898.92	895.64 to 902.20	<**0.001**
phenotype (plastic)	87.16	81.73 to 92.60	<**0.001**
transcriptome (sensitive)	925.47	921.81 to 929.13	<**0.001**
phenotype : transcriptome	55.78	50.07 to 61.49	<**0.001**
observations	8 055 548		
	no. of input–output exec instructions		
predictors	log-odd	CI (95%)	*p*-value
(intercept)	3.49	3.48 to 3.50	<**0.001**
phenotype (plastic)	0.68	0.67 to 0.69	<**0.001**
observations	487 705		

**Figure 4.**
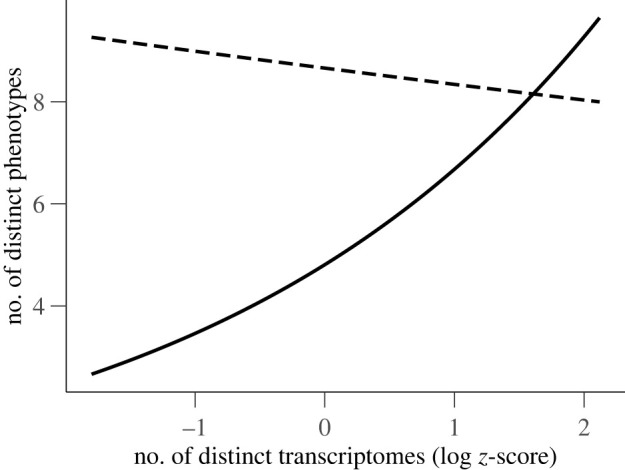
The effect of the cost to an organism’s viability on the relationship between phenotypic plasticity and sensitivity to changes in transcription. When phenotypic plasticity resulting from changes in the transcription of an organism’s genome comes at a cost to the organism’s viability, the higher is the number of distinct transcriptomes executed in different environments and the higher is the number of distinct phenotypes encoded by the genome in those environments (solid line). By contrast, when there are no costs, the higher is the number of distinct transcriptomes and the lower is the number of distinct phenotypes (dashed line). Only predicted lines of a zero-truncated negative binomial regression model are shown (*n* = 80 333).

Let us focus now on the genetic basis of phenotypic plasticity. We explored first to what extent the effect of the sensitivity of a genome to changes in transcription on plasticity is mediated by the cost to an organism’s viability. The number of distinct phenotypes at the average number of distinct transcriptomes was 75% higher when there were no costs on viability than when there were costs (e^2.15^ = 8.6 and e^2.15−0.56^ = 4.9, respectively; table 2). Moreover, when there were no costs, the number of distinct transcriptomes executed in different environments was higher and the number of distinct phenotypes encoded by the genome in those environments was lower. Specifically, a 1 s.d. increase in the logarithm of the number of distinct transcriptomes decreased the number of distinct phenotypes by 4% (from e^2.15^ = 8.6 to e^2.15−0.04^ = 8.2; figure 4). By contrast, when there were costs on viability associated with changes in transcription, a 1 s.d. increase in the logarithm of the number of distinct transcriptomes increased the number of distinct phenotypes by 29% (from e^2.150−0.56^ = 4.9 to e^2.15−0.56−0.04+0.33^ = 6.5; figure 4). Second, the sensitivity of an organism’s genome to changes in transcription mediated by the environment has a larger effect than plasticity on fitness (i.e. the length of the transcriptome was almost an order of magnitude larger: ±477 and ±58 instructions, respectively, for an average length of 1419 instructions; see electronic supplementary material). Specifically, for plastic organisms, environmentally sensitive transcriptomes were twice as long (i.e. higher fitness cost) than environmentally robust transcriptomes (899 + 87 + 925 + 56 = 1967, and 899 + 87 = 986 instructions, respectively; table 2). Third, the tandem repeats identified within transcriptomes (i.e. short sequences of instructions that were recurrently executed within a finite loop) contained *input–output* instructions that were executed twice as much when plasticity came from a genetical-based mechanism than when the mechanism for plasticity has no genetic basis (i.e. the average number of *input–output* instructions executed increased from e^3.49^ = 33 to e^4.17^ = 65; table 2). Finally, the uncertainty of computing a Boolean operation in a randomly chosen environment—measured as the Shannon entropy—was lower for organisms executing sensitive transcriptomes than robust ones (1/(1 + e^−2.3+1.43^) = 0.7 and 1/(1 + e^−2.3^) = 0.9, respectively, within a range between 0 and 1; table 1).

## 4. Discussion

Phenotypic plasticity is not only a pervasive feature of biological organisms but also of digital ones (figure 3). The environment can change the phenotype of digital organism by just providing different inputs to the same sequence of executed instructions. But it can also change the phenotype through a complex underlying genetic mechanism—by modifying the sequence of instructions that is actually executed (i.e. an organism’s transcriptome; figure 2). This genetic based pathway for plasticity comes at a cost to an organism’s viability and fitness. When that happens, the changes in transcription induced by the environment are so high they result in large phenotypic plasticity (figure 4). This trade-off between genetic-based plasticity and fitness cost can be explained by the higher number of *input–output* instructions executed within the tandem repeats located along the transcriptomes of plastic organisms: the longer the transcriptome, the more *input–output* instructions executed, and the higher the chances for the genome to encode novel phenotypes. Yet, changes in transcription decrease the uncertainty of the logic operations computed by an organism in a particular environment, regardless of the cost in viability and fitness. That is, the genomes of sensitive-transcriptome organisms encode many distinct phenotypes comprising almost the same logic operations. Moreover, changes in transcription reduce fitness in the absence of any selection pressure other than shortening the number of instructions that must be executed to produce a viable offspring.

In spite of the prevalence of phenotypic plasticity when digital organisms experience a wide range of environmental conditions, adaptation by natural selection might erode plasticity in evolving populations. Understanding the source of phenotypic plasticity (genetic or non-genetic) that would prevail under natural selection is out of the scope of this paper. Yet, the selective pressures that could favour one type of plasticity over the other were explored in a previous work [33] where the simplest strategy for selection was to evolve plasticity by putting different input numbers into the same execution flow. This plasticity with no genetic basis, which is analogous to the effect of the temperature on the phenotype (e.g. nearly all enzyme activity is temperature-dependent), comes at no fitness cost to the organisms. Changing the execution flow in response to the environment required, however, much more complex selective pressures. This is so because evolving populations store information about current and past environments [34]. My findings on the costs to an organism’s viability and fitness (i.e. longer replication times for sensitive-transcriptome organisms) suggest that selection will favour plasticity resulting from genetic-based mechanisms in large populations evolving in gradually changing environments that might promote directional selection. By contrast, in small populations evolving in unpredictable environments that might promote disruptive selection, plastic organisms resulting from executing the same transcriptome in different environments will be more fit than those who change their transcriptomes in response to changes in the environment. My expectations are based on the role of population size in counterbalancing the viability costs (i.e. a large population size reduces the extinction risk [35]), the ability to better cope with a gradual environmental change by tracing a smooth reaction norm [36], and the likely match between the directional selection and the constrained disparity of the polyphenisms [37].

The difficulty of disentangling the costs of plasticity from the costs of phenotype might explain why plasticity costs have been found to be negligible in most studies [25] (e.g. the fitness cost of the regulatory genetic machinery will typically be small). This is so because the cost of plasticity might be mitigated by the selective benefit of having the fittest phenotype. By contrast, the ability of quantifying fitness in the absence of any selective pressure favouring one phenotype over another in digital organisms allows us to detect unequivocally the cost of plasticity. Therefore, I suggest that the complications involved in detecting phenotypic costs in biological organisms can explain why plasticity costs have been detected in a few studies but found to be negligible in many others.

When two plastic genotypes produce the same phenotype in a particular environment, the organism sensitive to changes in transcription might have reduced fitness compared with the robust-transcriptome organism because of its lower viability and longer generation time. The costs in viability found when plasticity results from genetic-based mechanisms might be analogous to the developmental instability leading to a reduction in the performance traits that can result in low fitness [26]. On the other hand, the fitness cost of having longer generation times might be analogous to the cost of phenotype production in biological organisms. Specifically, increasing the transcriptome structural complexity (i.e. increasing the length and number of input–output instructions executed) might be considered analogous to increasing the energetic cost of gene replication, maintenance, and expression in biological organisms. There are several transcription-associated costs—measured in ATP units—in biological organisms, such as investments in the synthesis of ribonucleotides, turnover of transcripts within the lifespan of the cell, and activation and initiation of transcription. These costs are relevant because the long-term preservation of a gene by natural selection requires that its phenotypic benefits exceed the energetic costs to a large enough extent to offset the power of random genetic drift [38].

The potential of digital organisms to carry out studies on plasticity allows direct testing of theoretical models, particularly the prediction that variable versus static environments indeed select for or against plasticity, respectively. It is also possible to obtain insights to make a parallelism on, for example, the genes, sensory proteins, enzymes, and biochemical pathways—in short, the molecular machinery responsible for phenotypic plasticity. In fact, the two mechanisms for phenotypic plasticity reported here, analogous to the developmental process of biological organisms, support, once more, the parallelism between artificial and natural evolving systems. Simultaneously, empirical data about population sizes or mutation rates in digital organisms make it possible to address the effect of selection, mutation, and genetic drift on the maintenance or loss of plasticity and the associated mechanisms regulating that plasticity.

Phenotypic plasticity can eventually be lost through genetic assimilation. Genetic assimilation occurs when a trait that was originally triggered by the environment loses this environmental sensitivity and ultimately becomes fixed or expressed in a population [10,39]. This process might promote the origins of novel traits and possibly fuel speciation and adaptive radiation. In digital organisms genetic assimilation might also take place, specifically, if selection drives phenotypic plasticity through changes in transcription. This is so because cryptic genetic changes (i.e. mutations that do not change the phenotype) are more likely to pave the way to adaptive mutations encoding a novel phenotype that match a gradual environmental change, as modifications in transcription trace a smooth reaction norm [36]. In contrast to genetic mutations, which initially affect only one organism, environmentally induced novelties are likely to have a huge evolutionary potential because changes in the environment impact many organisms simultaneously. Therefore, plasticity provides fertile ground for selection to act and increases the chances that genetic assimilation will occur [40].

Lastly, phenotypic plasticity plays a key role in the genotype-phenotype map. Polyphenisms in digital organisms increase the overlap between genotype networks because plastic genotypes will act as bridges connecting non-plastic genotypes encoding different phenotypes [31,41]. That is, the boundaries between genotype networks are no longer uniquely defined, but depend explicitly on the environment.

## 5. Future directions

This work on the phenotypic plasticity of digital organisms opens up new avenues for further research in many areas of evolutionary biology. Future studies will shed light on the role of phenotypic plasticity in promoting or hindering adaptation, characterizing the genotype–phenotype map, fostering the navigability of fitness landscapes, shaping the relationship between robustness and evolvability, promoting genetic assimilation, driving demographic changes of invasive species, and accelerating the rate of coevolution.

## Data Availability

The data reported in this article and the code to analyse the data are available on GitHub (https://gitlab.com/fortunalab/publications/phenotypic-plasticity-of-an-evolving-digital-organism) and have been archived on Zenodo: http://dx.doi.org/10.5281/zenodo.6501535 [42].
